# High Mobility Group Box 1 Protein as a Biomarker in Adult Attention Deficit Hyperactivity Disorder

**DOI:** 10.31083/AP46947

**Published:** 2025-09-26

**Authors:** Gözde Yontar, Selim Görgün

**Affiliations:** ^1^Department of Psychiatry, Samsun Training and Research Hospital, 55200 Samsun, Turkey; ^2^Department of Microbiology and Clinical Microbiology Laboratory, Samsun Training and Research Hospital, 55200 Samsun, Turkey

**Keywords:** attention deficit hyperactivity disorder, High Mobility Group Box 1 protein, inflammation, neurodevelopment

## Abstract

**Background and Objectives:**

Attention deficit hyperactivity disorder (ADHD), while representing the most frequently diagnosed and treated condition in child and adolescent psychiatry, continues to be underrecognized and inadequately managed in adult populations. Numerous studies have explored how ADHD may be connected to the immune system and inflammatory processes. These studies have focused particularly on ADHD, stress, anxiety and immune dysregulation. High Mobility Group Box 1 (HMGB1), a nuclear transcription factor and a late-phase mediator of inflammation, has been found to be elevated in various neuropsychiatric conditions. This study aimed to elucidate the potential contribution of inflammatory mechanisms to the pathophysiology of ADHD by quantifying HMGB1 levels.

**Materials and Methods:**

43 ADHD patients and 42 controls with an age between 18–65 years were enrolled. Patients with any acute or chronic psychiatric disease, chronic inflammatory or autoimmune disease, substance addiction, malignancy, severe systemic disease, schizophrenia, mental retardation, a history of surgery or head trauma in the last 6 months and who were on vitamin or fish oil supplements or steroids were excluded. Blood samples were obtained and HMGB1 was measured with Enzyme-Linked Immuno Sorbent Assay method.

**Results:**

The two groups exhibited comparable sociodemographic characteristics. HMGB1 levels were significantly higher in ADHD group than controls (967.5 ± 462.0 ng/mL vs 693.4 ± 366.9 ng/mL, *p* = 0.003).

**Conclusion:**

In our study, the finding that HMGB1 serum levels were higher in adult ADHD patients compared to healthy controls supports the hypothesis that chronic low-grade inflammation, which is both driven and detected by HMGB1, may be associated with ADHD through the possibility of causing neurodevelopmental disorders. It is known that HMGB1 is effective in the diagnosis and prognosis of immune system diseases. Therefore, our results show that HMGB1 may be related to the pathophysiology of ADHD.

## Main Points

1. Attention deficit hyperactivity disorder (ADHD) and Inflammation Connection

The study investigates the potential inflammatory basis of Adult ADHD, focusing on High Mobility Group Box 1 (HMGB1)—a protein known to be a pro-inflammatory mediator and danger-associated molecular pattern (DAMP). While previous studies have linked ADHD with autoimmune and inflammatory disorders, the role of HMGB1 in ADHD had not yet been explored.

2. Study Design and Participant Selection

The study included 85 participants (43 with adult ADHD, 42 healthy controls), matched for age, sex, socioeconomic status, smoking, and body mass index (BMI). Strict exclusion criteria were applied to eliminate confounding factors, such as infections, autoimmune diseases, psychiatric comorbidities, substance use, and recent surgeries or supplement intake.

3. Key Finding: Elevated HMGB1 in ADHD

Serum HMGB1 levels were significantly higher in the ADHD group (967.5 ± 462.0 ng/mL) than in the control group (693.4 ± 366.9 ng/mL), with *p* = 0.003, suggesting a potential role for chronic low-grade inflammation in the pathophysiology of adult ADHD.

4. Validated Diagnostic and Screening Tools Used

Participants were assessed using the Diagnostic and Statistical Manual of Mental Disorders, Fifth Edition (DSM-5) criteria, Structured Clinical Interview for DSM Disorders (SCID)-5 ADHD module, Adult ADHD Self-Report Scale (ASRS), and Wender Utah Rating Scale (WURS). These tools showed strong validity and reliability for evaluating adult ADHD and ensuring diagnostic consistency.

5. Implications and Future Directions

This is the first study to demonstrate elevated HMGB1 levels in adults with ADHD, indicating that HMGB1 may serve as both a biomarker and potential mechanistic contributor to neuroinflammatory processes in ADHD. Further research is needed to confirm causality and explore HMGB1-targeted interventions.

## 1. Introduction

Among neurodevelopmental disorders, attention deficit hyperactivity disorder (ADHD) stands as the most commonly identified and managed condition in child and adolescent psychiatric practice [1]. ADHD is reported to have prevalence of 2.5–4.9% in the general adult population [2]. ADHD is still not sufficiently recognized in adult psychiatry, it is overlooked, and cases that apply to psychiatry for the treatment of ADHD-related problems are treated with other diagnoses [3, 4]. For these reasons, it is estimated that 90% of adults with ADHD remain untreated [5].

There are many studies investigating the relationship between ADHD, the immune system and inflammation. These studies have focused particularly on ADHD, stress, anxiety and immune dysregulation. Undoubtedly, the presence of ADHD symptoms causes patients to experience many conflicts, neglect, physical and emotional abuse in their family, school and social lives [6, 7]. The probability of children with ADHD experiencing head trauma and physical trauma is also quite high, and this can be considered as another stressor for them [8, 9]. Evidence from cross-sectional observational studies, systematic reviews, and meta-analyses indicates a significant association between ADHD and various autoimmune and inflammatory conditions. These encompass a broad spectrum of conditions that impact neuroinflammatory and neurodevelopmental processes, including diabetes mellitus, psoriasis, allergic rhinitis and conjunctivitis, atopic dermatitis, maternal autoimmune diseases, prenatal stress, microbiome-related inflammation, polymorphisms in inflammation-associated genes, and alterations in immunological markers [10, 11, 12]. However, the role of inflammation in ADHD has not been fully explained. The identification of biological markers that inform the diagnosis, treatment, and prognosis of ADHD remains limited. Consequently, there is a growing need for novel etiological models to elucidate the underlying pathophysiological mechanisms of the disorder. In recent years, several studies have examined the role of High Mobility Group Box 1 (HMGB1) in various neuropsychiatric conditions. HMGB1, a nuclear transcription factor, functions as a late-phase mediator of inflammation and may offer insights into the inflammatory processes implicated in ADHD. The 30 kilodaltons molecular weight protein was released from the cell nucleus together with histones and was named “High Mobility Group Box 1” due to its rapid movement in the electrophoresis gel [13, 14]. HMGB1 is found in the cytoplasm of liver and brain cells, while it is found in both the cytoplasm and nucleus of lymphoid cells [15]. HMGB1 plays a role as an extracellular signaling molecule in inflammation, cell differentiation, cell migration and tumor metastasis [16]. HMGB1 released from activated macrophages can play a role in immune activity as a cytokine by activating other cells involved in the immune response [17]. HMGB1 is thought to have an important role in the activation of proinflammatory markers such as Tumor Necrosis Factor (TNF)-a, Interleukin (IL)-1b and IL-8 [18, 19]. HMGB1 passively released from necrotic and damaged cells is responsible for carrying the damage signal to neighboring immune system cells. HMGB1, released from cells undergoing necrosis, plays a role in the circulation as the Danger Associated Molecular Pattern (DAMP) protein, activating immune cells and increasing phagocytosis [16, 19, 20]. HMGB1 is recognized as a critical mediator of chronic low-grade inflammation, exerting its effects by acting as a DAMP molecule that activates immune responses through receptors such as receptor for advanced glycation end-products (RAGE) and Toll-like receptors (TLRs) [17, 19]. As a result, HMGB1 plays a role in the diagnosis and course of immune system diseases. Importantly, HMGB1 is not only a marker of inflammation but also a potent mediator that can amplify and sustain inflammatory responses, particularly within the central nervous system. Its role has been studied in various neuropsychiatric and neurodevelopmental conditions, including autism spectrum disorder and major depressive disorder, suggesting its broader relevance to brain-immune interactions [21, 22]. Given this dual function as a biomarker and active contributor to inflammation-related neuronal dysregulation, HMGB1 represents a compelling target for exploration in ADHD. There is no study in the literature examining the level of HMGB1 in adult ADHD. The aim of our study is to explore the role of HMGB1, not only as a potential marker but also as a possible mediator of inflammation in the pathophysiology of ADHD, by comparing HMGB1 levels in patients with ADHD and healthy controls.

## 2. Methods and Materials

### 2.1 Patient Enrollment

Individuals diagnosed with adult ADHD based on Diagnostic and Statistical Manual of Mental Disorders, Fifth Edition (DSM-5) criteria, who presented to the Psychiatry Outpatient Clinic of Samsun Training and Research Hospital between December 2024 and February 2025, were enrolled in the study. The control group was composed of individuals without any documented history of psychiatric or significant medical conditions.

Participant exclusion from the study was based on the subsequent criteria.

Individuals who were younger than 18 or older than 65 years old, were diagnosed with depressive disorder, anxiety disorder, inflammatory or autoimmune disease, active infection, pregnancy, severe systemic disease (epilepsy, diabetes, liver failure, kidney failure, hypertension, heart diseases), alcohol addiction, substance addiction, schizophrenia. Those who had C-reactive protein (CRP) serum level higher than 3 milligram/Liter at the time of enrollment, those with chronic psychotic diseases such as schizophrenia, those with a history of severe head trauma, alcohol addiction, substance addiction, those with mental retardation, those who have used corticosteroids, fish oil supplements, vitamin supplements or drugs that affect the immune system in the last 6 months, those with malignancy, those with a history of surgery in the last 6 months.

After the participants signed the informed consent form, the sociodemographic information form and the Adult Attention Deficit Hyperactivity Disorder Self-Report Scale (ASRS-1), Adult Attention Deficit Hyperactivity Disorder Wender Utah Rating Scale (WURS) were applied. Blood was collected for biochemical analysis.

A priori power analysis was conducted using G*Power v3.1.9.6 (https://www.psychologie.hhu.de/arbeitsgruppen/allgemeine-psychologie-und-arbeitspsychologie/gpower) to determine the required sample size based on the study’s primary outcome: the difference in serum HMGB1 levels between adults with ADHD and healthy controls. A two-tailed independent-samples *t*-test was selected as the appropriate method, given the case-control design and the continuous nature of the HMGB1 variable. Assuming a medium effect size (Cohen’s d = 0.6) based on findings from prior studies on inflammatory biomarkers in neuropsychiatric conditions, and setting the alpha level at 0.05 with a desired power of 95%, the required sample size was calculated to be 84 participants (42 per group). A slightly higher power threshold (95% rather than 80%) was selected to minimize the risk of Type II error given the exploratory and clinically meaningful role of HMGB1 as a potential biomarker. Accordingly, 85 participants were enrolled (43 ADHD, 42 controls), meeting the required sample size based on this a priori calculation. Volunteers admitted to the hospital for routine health evaluations (e.g., job applications, university registration) and matched with the patient group by age and sex were included in the control group. After exclusions, 42 individuals were deemed eligible to participate in the study.

### 2.2 Data Collection Tools

#### 2.2.1 Participant Background Information

Participants provided personal information and data on specified variables through a sociodemographic form created by the researcher. Participants reported their monthly household income, and their socioeconomic status was classified based on the 2024 income thresholds defined by the Turkish Statistical Institute [23]. For privacy purposes, participants were asked to identify themselves using pseudonyms instead of their real names.

#### 2.2.2 Structured Clinical Interview DSM-V Axis 1 Disorders (SCID-1)

A semi-structured clinical interview tool, the Structured Clinical Interview DSM-V Axis 1 Disorders (SCID-1), is utilized for the diagnosis of primary Axis 1 disorders [24]. The Turkish adaptation and reliability of the scale were assessed by Elbir and colleagues [25]. In this study, the ADHD module of the Turkish version was administered by trained clinicians to assess the presence of Adult ADHD. The module evaluates inattention and hyperactivity/impulsivity symptoms based on DSM-5 criteria, and a diagnosis requires at least five symptoms from either or both domains, with evidence of onset before age 12 and clinically significant impairment across settings. The SCID-5 showed outstanding inter-rater reliability in assessing ADHD, as evidenced by a Cohen’s kappa coefficient of 1.00. This perfect level of agreement between raters supports the tool’s consistency and suitability for diagnosing ADHD among adults in the Turkish population.

#### 2.2.3 Adult Attention Deficit Hyperactivity Disorder Self-Report Scale (ASRS)

It is a scale developed by the World Health Organization for the screening of ADHD [26]. The validity and reliability study in Turkish was conducted by Doğan *et al*. [27]. The adult ADHD Self-Report Scale (ASRS-v1.1) is an 18-item self-report tool used to assess adult ADHD symptoms, with two subscales: Inattention and Hyperactivity/Impulsivity, each containing 9 items. Responses are rated on a 5-point Likert scale (0 = never, 4 = very often). While the 6-item screener uses a cut-off of 4 or more positive responses, the full 18-item version is interpreted based on clinical judgment rather than a strict cut-off score. The Turkish adaptation of the ASRS-v1.1 demonstrated robust psychometric properties. Internal consistency was high, with a Cronbach’s alpha of 0.88 for the overall scale, 0.82 for the Inattention subscale, and 0.78 for the Hyperactivity/Impulsivity subscale. Test-retest reliability was also satisfactory, showing a correlation coefficient of r = 0.85 for the total score. Factor analysis supported the bifactor structure of the scale, accounting for 41.6% of the total variance. These findings confirm that the Turkish version of the ASRS-v1.1 is both valid and reliable for assessing adult ADHD symptoms.

#### 2.2.4 Adult Attention Deficit Hyperactivity Disorder Wender Utah Rating Scale (WURS)

It was developed in 1993 by the Utah group of Wender and Reimherr to assess the childhood symptoms and findings of adults related to ADHD. The Wender Utah Rating Scale (WURS) is a 25-item retrospective self-report instrument used to assess childhood symptoms of ADHD in adults. The Turkish adaptation of this scale was evaluated for its validity and reliability in a study involving 59 adults diagnosed with ADHD, 59 with depression, 44 with bipolar disorder in remission, and 145 healthy controls. The scale showed strong internal consistency, with a Cronbach’s alpha of 0.93, and demonstrated good test-retest reliability (r = 0.81). Exploratory factor analysis identified five distinct dimensions—irritability, depressive symptoms, academic difficulties, impulsive and behavioral concerns, and attention-related issues—which together explained 61.3% of the total variance. Using a cut-off score of 36, the tool correctly classified 82.5% of individuals with ADHD and 90.8% of those in the control group. Nonetheless, symptom overlap with mood disorders may limit the scale’s specificity in differential diagnosis [28].

#### 2.2.5 Blood Analysis and Indicator Assessment

In our study, blood samples were obtained during routine blood panel work-up for the patients/clients at the psychiatric outpatient clinic. Outpatient blood samples, collected electively, were obtained with consideration for fasting status and timing. A 5 mL venous blood sample was drawn from each participant and centrifuged at 4000 rpm for 10 minutes. The separated serum was transferred into dedicated storage tubes and preserved at –80 °C until analysis, which was conducted using the enzyme-linked immunosorbent assay (ELISA) technique. Human HMGB1 ELISA Kit (Fine Test, Catalogue no: EH0884, UniProt ID: P09429, Wuhan, Hubei, China) was used in this procedure. The results read at 450 nm and calculated by Infinite (version: 200 PRO, serial number: 1503007387, Tecan, Grödig, Austria).

### 2.3 Statistical Analysis

Statistical analyses were conducted using IBM SPSS Statistics for Windows, version 21.0 (IBM Corp., Armonk, NY, USA). The distribution of continuous variables was assessed using the Kolmogorov-Smirnov test. Based on the distributional characteristics, independent samples *t*-tests or Mann-Whitney U tests were applied to compare continuous variables. Categorical variables were analyzed using either the Chi-square test or Fisher’s exact test, as appropriate. Continuous data are presented as mean ± standard deviation (SD), while categorical data are expressed as frequencies and percentages. To assess the association between independent variables and the presence of ADHD, a binary logistic regression analysis was conducted. Both univariate and multivariate models were used to evaluate the potential predictors of ADHD. Additionally, the diagnostic performance of the HMGB1 parameter in predicting ADHD was examined using Receiver Operating Characteristic (ROC) curve analysis. The area under the curve (AUC), optimal cut-off value, sensitivity, specificity, positive predictive value (PPV), and negative predictive value (NPV) were calculated to assess the discriminative ability of HMGB1 levels. Statistical significance was defined as a *p*-value below 0.05.

## 3. Results

A total of 85 (n = 43 for ADHD group, n = 42 for controls) patients were enrolled. The two groups were comparable in terms of sociodemographic variables, including age, relationship situation, income level, occupational situation, and educational attainment (Table 1). The study sample primarily included young to middle-aged adults, with a mean age of 40.9 ± 13.3 years. The majority of participants were male (n = 66, 77.6%), with no statistically significant difference in gender distribution between groups. Approximately three-quarters of individuals in both groups were employed. Both groups had similar amounts of low- and middle-income individuals and very few with high income. It is possible that study group was reflecting low and middle class of society. Individuals classified as heavy smokers were excluded from the study, and the proportion of light smokers did not differ significantly between the two groups. Both groups were significantly differed in terms of ASRS and WURS scores, as expected (Table 2). HMGB1 levels were compared between the ADHD and control groups. The mean HMGB1 concentration in the ADHD group (967.5 ± 462.0 ng/mL) was significantly higher than that observed in the control group (693.4 ± 366.9 ng/mL), with the difference reaching statistical significance (*p* = 0.003), as shown in Table 2. HMGB1 levels of smokers and non-smokers in the patient group were evaluated. It was found that the HMGB1 levels of smokers (934.9 ± 373.3 ng/mL) and non-smokers (1008.7 ± 562.7 ng/mL) in the ADHD group were not statistically significantly different (*p* = 0.626).

**Table 1.** **Participant demographics**.

Parameter		ADHD group (n = 43)	Control group (n = 42)	*p* value
Age, years		38.6 ± 12.4	43.3 ± 13.9	0.104
Female sex (n, %)		10 (23.2%)	9 (21.4%)	0.840
Working status				
	Employed (n, %)	30 (69.7%)	33 (78.5%)	0.354
Marital status				
	Single/divorced (n, %)	22 (51.1%)	18 (42.8%)	0.443
Educational degree				
	Middle school (n, %)	5 (11.6%)	7 (16.6%)	0.798
	High school (n, %)	21 (48.8%)	19 (45.2%)	
	University or higher (n, %)	17 (39.5%)	16 (38.0%)	
Financial status				
	Low income (n, %)	18 (41.9%)	18 (42.9%)	0.840
	Middle income (n, %)	18 (41.9%)	19 (45.2%)	
	High income (n, %)	7 (16.2%)	5 (11.9%)	
Smoker (n, %)		24 (55.8%)	19 (45.2%)	0.330
Body mass index		21.9 ± 1.8	22.0 ± 2.0	0.790

ADHD, attention deficit hyperactivity disorder, a *p* value < 0.05 is accepted to be of significance.

**Table 2.** **Participants’ biochemical and hematological results, with ASRS and WURS scores**.

Parameter	ADHD group (n = 43)	Control group (n = 42)	*p* value
HMGB1 (ng/mL)	967.5 ± 462.0	693.4 ± 366.9	0.003
Hb (g/L)	15.1 ± 1.3	14.9 ± 1.4	0.408
Neu (10^9^/L)	4.5 ± 1.3	4.6 ± 1.5	0.781
Lym (10^9^/L)	3.3 ± 0.8	3.4 ± 0.8	0.672
Plt (10^9^/L)	291.3 ± 95.1	307.9 ± 92.5	0.418
Bun (mg/dL)	17.8 ± 4.9	16.7 ± 5.2	0.337
Creatinine (mg/dL)	1.2 ± 0.3	1.3 ± 0.3	0.128
AST (mg/dL)	21.7 ± 6.0	20.7 ± 5.5	0.405
ALT (mg/dL)	25.0 ± 9.2	25.5 ± 8.6	0.806
ASRS score (points)	29.9 ± 2.7	7.6 ± 2.3	<0.001
WURS score (points)	56.5 ± 8.9	17.4 ± 7.9	<0.001

ADHD, Attention deficit hyperactivity disorder; ASRS, Adult Attention Deficit Hyperactivity Disorder Self-Report Scale; AST, aspartate transaminase; ALT, alanine transaminase; BUN, blood urea nitrogen; g, gram; Hb, hemoglobin; HMGB-1, high mobility group box 1 protein; Lym, lymphocyte; L, liter; mg, milligram; mL, milliliter; ng, nanogram; neu, neutrophil; plt, platelet; WURS, Adult Attention Deficit Hyperactivity Disorder Wender Utah Rating Scale, a *p *value < 0.05 is accepted to be of significance.

Binary logistic regression analysis was used to evaluate the impact of independent variables on ADHD, considering both univariate and multivariate models (Table 3). The findings indicated that HMGB1 levels were significantly associated with ADHD in both models. Specifically, each one-unit increase in HMGB1 was linked to a 1.002-fold rise in the likelihood of having ADHD, with *p*-values of 0.006 in the univariate model and 0.029 in the multivariate model. No other variables demonstrated a statistically significant association (*p* > 0.05).

**Table 3.** **Evaluation of the association between independent variables and ADHD using binary logistic regression**.

Variable	Univariate		Multivariate	
	OR (95% CI)	*p*	OR (95% CI)	*p*
Gender (Ref: Male)	1.111 (0.400–3.086)	0.840	2.357 (0.427–13.006)	0.325
Smoker (Ref: Non-smoker)	1.529 (0.650–3.596)	0.330	1.341 (0.321–5.598)	0.687
Employment status (Ref: Unemployed)	0.629 (0.235–1.682)	0.356	0.533 (0.099–2.863)	0.463
Marital status (Ref: Single)	0.716 (0.304–1.683)	0.444	0.731 (0.137–3.893)	0.713
Education status (Ref: Middle school)	Reference			
High school	1.326 (0.348–5.062)	0.679	7.372 (0.556–97.716)	0.130
University or higher	1.457 (0.366–5.801)	0.593	3.114 (0.287–33.783)	0.350
Financial status (Ref: Low)	Reference			
Middle	0.848 (0.338–2.124)	0.724	2.352 (0.499–11.082)	0.280
High	1.326 (0.356–4.947)	0.674	1.909 (0.210–17.313)	0.566
Age	0.978 (0.943–1.013)	0.218	0.996 (0.931–1.065)	0.905
HMGB1	1.002 (1.000–1.003)	0.006	1.002 (1.000–1.003)	0.029
Hb	1.141 (0.837–1.555)	0.403	0.953 (0.622–1.460)	0.826
Neu	0.959 (0.715–1.286)	0.778	0.862 (0.552–1.348)	0.515
Leu	0.896 (0.542–1.480)	0.668	1.137 (0.548–2.362)	0.730
Plt	0.998 (0.994–1.003)	0.413	0.998 (0.991–1.005)	0.573
BUN	1.043 (0.958–1.134)	0.332	1.058 (0.935–1.196)	0.371
Creatinine	0.868 (0.288–2.617)	0.802	0.428 (0.077–2.387)	0.333
AST	1.032 (0.958–1.112)	0.400	1.032 (0.932–1.143)	0.543
ALT	0.994 (0.947–1.043)	0.804	1.005 (0.943–1.071)	0.873

A *p* value < 0.05 is accepted to be of significance.

**Fig. 1.**
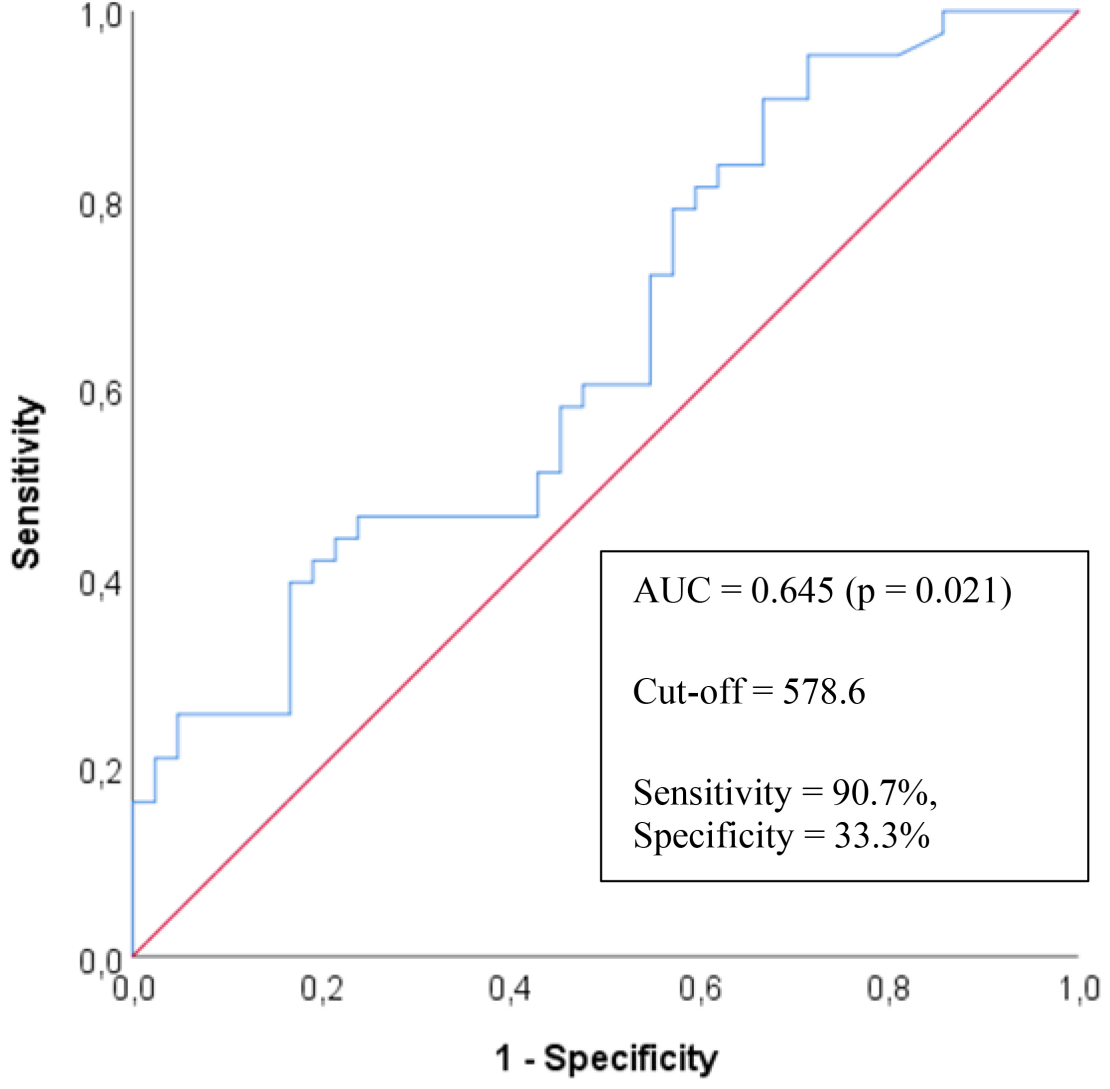
**ROC curve for serum High mobility group box 1 protein levels in predicting attention deficit hyperactivity disorder**.

The AUC value for the HMGB1 parameter in predicting ADHD was calculated as 0.645, indicating a statistically significant result (*p* = 0.021) (Table 4). When the cut-off value was set at 578.6 ng/mL, the model demonstrated a sensitivity of 90.7%, specificity of 33.3%, PPV of 58.21%, and NPV of 77.78% (Fig. 1).

**Table 4.** **ROC Analysis of the HMGB1 Parameter in Predicting ADHD**.

	AUC (95% CI)	*p*	Cutpoint	Sensitivity (%)	Specificity (%)	PPV (%)	NPV (%)
HMGB1	0.645 (0.528–0.762)	0.021	578.6	90.70	33.33	58.21	77.78

A *p* value < 0.05 is accepted to be of significance. ROC, Receiver Operating Characteristic; AUC, area under the curve; PPV, positive predictive value; NPV, negative predictive value.

## 4. Discussion

In our study, serum HMGB1 levels of ADHD patients and healthy controls were examined. The main finding of our study was that serum HMGB1 levels were significantly higher in ADHD patients than in healthy controls. To the best of our knowledge, no previous studies have directly compared serum HMGB1 levels between ADHD patients and healthy controls. Consequently, our study represents the first investigation into this potential relationship.

ADHD is the most prevalent neurodevelopmental disorder of childhood, with approximately three-quarters of cases persisting into adulthood. Given its chronic nature and wide-ranging impact, ADHD is increasingly recognized as a significant public health concern [29]. ADHD is a disease characterized by attention deficit, hyperactivity, impulsivity. In addition, low school performance, sleep disorders, learning disabilities, anxiety disorders, mood disorders, tics, oppositional defiant disorder, and behavioral disorders are the main findings seen in ADHD, independent of socioeconomic factors [30, 31]. In addition to all these, ADHD is also strongly associated with emotional dysregulation. Irritability, poor anger control, anxiety, deficiencies in establishing and maintaining social relationships, emotional instability, dysphoria are prominent symptoms of emotional dysregulation [32, 33, 34].

The underlying mechanisms that cause ADHD development have not yet been sufficiently elucidated. However, decreased volume and loss of function in the gray and white matter of the brain have been associated with impaired attention, cognition, processing speed, motor planning, and increased behavioral problems [35, 36]. Many studies have drawn attention to deficits associated with the cerebellum, caudate, and prefrontal cortex for ADHD. These areas of the brain have neuronal structures that regulate attention, movement, behavior, emotions, and thoughts [37, 38]. Plasma monoamine levels have been measured in many studies to explain the underlying biological mechanisms in ADHD. In particular, it has been hypothesized that norepinephrine and dopamine neurotransmitters mediate communication in the pre-synaptic and post-synaptic spaces of neuronal networks in the mentioned brain areas, and that catecholamines are hypo- and hyperactive in these regions in ADHD [37, 39, 40]. In studies conducted in children, it has been thought that neurodevelopmental disorders caused by neuroinflammation are caused by microglial changes, astrocytes, chemokine and cytokine release and oxidative stress [41]. However, it is thought that material inflammation and immune system dysfunction in children also increase the risk of ADHD [42]. Gustafsson *et al*. [43] drew attention to the risk between high serum IL-6, TNF-a and monoxide chemoattractant protein-1 detected in material serum and the development of ADHD and was the first study to examine the relationship between inflammation and brain development and behavioral deficits. Many animal studies have supported the development of ADHD in the offspring by maternal immune system activation [44]. It has been determined that conditions associated with high inflammation, such as exposure to heavy metals in the perinatal period, which may affect neurodevelopmental mechanisms, also increase the risk of ADHD [45]. Peripheral pro-inflammatory cytokines pass to humoral and neural pathways in the brain, causing inflammatory responses in the neuroimmune system. Elevations in inflammation-related cytokines within the brain have been shown to induce alterations in dopaminergic pathways, which resemble those observed in ADHD. Furthermore, exposure to heightened inflammatory levels during the prenatal period can result in lasting changes to neuronal circuits, as well as structural abnormalities in gray matter volume [46]. In addition, chronic cytokine release caused by the hypothalamo-pituitary axis has been accepted as another risk factor associated with the development of ADHD [47]. Chang* et al*. [48, 49] compared adult ADHD patients with healthy controls and found that CRP levels in the blood were high in ADHD patients. Oades [45] also found that serum IL-1B, IL-6, IL-10, IL-13, IL-16 and TNF-a levels were significantly higher in the ADHD group than in the control group [45]. However, findings across studies vary, and the exact nature and clinical significance of these differences remain an area of ongoing investigation.

HMGB1, known as a nuclear transcription factor, has also been reported to be a late indicator of inflammation. It is thought that HMGB1 plays an important role in the activation of proinflammatory indicators TNF-a, IL-1B and IL-8. Therefore, HMGB1 plays a role in the course of immune system diseases, diagnosis and determination of disease prognosis. In addition, HMGB1 is a non-histone protein and has the ability to regulate DNA repair, transcription enhancement in the nucleus, cell differentiation, apoptosis and autophagy in the cytoplasm after cell damage [50, 51]. It acts as a proinflammatory cytokine [52] and also affects the release of proinflammatory cytokines/chemokines such as TNF, IL-1b or CXCL8 [53]. Since it triggers inflammation and disrupts the vascular barrier in tissues [54], it is a biological marker of neuroinflammation and neurodegeneration that can cause blood-brain barrier dysfunction [55]. This protein has been identified as a risk factor for the progression of inflammation within the nervous system in patients with Alzheimer’s disease, Parkinson’s disease, and multiple sclerosis [56]. Alterations in HMGB1 serum concentrations have been observed in children with autism spectrum disorder, with elevated levels reported. Additionally, it has been suggested that HMGB1’s neurotoxic effects may contribute to the neurocognitive impairments and various symptom clusters observed in schizophrenia [57].

Numerous studies have demonstrated that HMGB1 may inhibit autophagy within neurons. Autophagy plays a crucial role in maintaining neuronal health by clearing cellular debris and regulating the turnover of intracellular components. In neurons, which are particularly sensitive to disruptions in cellular homeostasis, impaired autophagic activity can lead to the accumulation of misfolded proteins and dysfunctional organelles. This disruption not only compromises neuronal function but also triggers apoptosis and contributes to neurodegenerative processes [58, 59]. To date, more than thirty mammalian autophagy genes have been identified that play an important role in the occurrence and regulation of the autophagy process. In addition to these proteins, the role of many proteins, including HMGB1, in the autophagy process has been investigated. It is involved in the regulation of autophagy with Beclin 1 in the cell cytoplasm. HMGB1 binds to Beclin 1, which plays a role in the regulation of autophagosome formation and maturation in autophagy, and induces autophagy [60]. Beclin 1, a protein in the regulation of autophagy, plays a role in the formation of autophagosomes [61], and a decrease in the level of Beclin 1 can trigger apoptotic processes [62]. Under normal conditions, basal autophagy in the central nervous system serves a protective role by clearing misfolded and damaged protein aggregates, thereby mitigating protein toxicity and oxidative stress, and supporting the longevity of neural cells. However, disturbances in autophagic processes—such as impaired autophagosome clearance or excessive autophagic activity—can have detrimental effects, potentially leading to neuronal cell death [63]. Beclin 1 is a protein molecule synthesized from neurons and glia [64], and is known to play a role in the pathogenesis of some heart diseases, Parkinson’s and Alzheimer’s diseases [65, 66, 67]. It has been stated that this protein may play a role in autism due to the disorder in the autophagy system [68]. In schizophrenia, disruptions in neuronal autophagy can result in cellular dysfunction, leading to widespread alterations across various brain regions that contribute to the manifestation of disease symptoms [69]. Reduced levels of Beclin 1 have been observed in the hippocampus of these patients, highlighting the critical role of this protein in the initiation of autophagy processes, which are closely linked to the onset of apoptosis [70]. It is known that HMGB1 is a new Beclin 1 binding protein that is important in maintaining autophagy [71]. It has been shown that it causes autophagy dysfunction by affecting Beclin 1 and as a result, causes neurotoxicity [72].

In the central nervous system, basal levels of autophagy play a protective role by clearing misfolded or damaged protein aggregates, thereby reducing protein toxicity and oxidative stress, and supporting the longevity of neural cells. However, when autophagic processes are disrupted—such as through the buildup of defective autophagosomes or overstimulation of autophagy—it can result in cellular dysfunction and ultimately trigger neuronal cell death [63].

## 5. Conclusions

In our study, elevated serum HMGB1 levels were observed in adult ADHD patients compared to healthy controls. This finding raises the possibility that HMGB1 may be involved in the neurobiological mechanisms associated with ADHD. Given HMGB1’s recognized relevance in the pathogenesis and prognosis of immune-related conditions, it may warrant further investigation as a potential biomarker in ADHD. Nevertheless, definitive conclusions cannot be drawn from the current data, and additional studies are necessary to elucidate the precise role of HMGB1 in the pathophysiology of ADHD. Building on these findings, exploring HMGB1 as a biomarker could have important clinical implications. A more comprehensive understanding of this mechanism could facilitate improved recognition and diagnosis of adult ADHD, a condition that is frequently underrecognized and mischaracterized. Identifying a quantifiable biological indicator may assist in diminishing stigma, supporting early intervention, and encouraging a more unified approach to mental and physical health. In the broader context, this study aims to advance public awareness and strengthen both the support and care available to adults managing ADHD.

## 6. Limitations

It is not possible to predict the direct relationship between high HMGB1 levels and neurodevelopmental disorders in ADHD in this study. This study has various strengths and limitations. When we look at the strengths of our study, it can be considered that the study consisted of a sample that excluded additional physical diseases and psychiatric disorders that may be associated with inflammation. However, it can be considered that the fact that the ADHD group consisted entirely of untreated patients increased the reliability of the results. There are some limitations to this study. First of all, our sample size was relatively small which is typical in experimental and hypothesis-driven biomarker research. While power analysis showed sufficient statistical power, the generalizability of our findings may be limited by the sample size. Another shortcoming was the absence of inflammatory markers other than HMGB1. On the other hand, our goal was to exclude possible “manifest” inflammation and provide room for searching merely “neural inflammation” which was driven and documented by increased HMGB1 levels. Authors thought that it would highlight HMGB1’s value to show “subtle neural low grade chronic inflammation” just because HMGB1 is found in the cytoplasm of liver and brain cells. In addition, authors suggest that HMGB1 may play a role in neurodevelopmental deficiency in ADHD. In this case, our study remains an experimental and hypothetical research. In future studies, it may be more appropriate to measure inflammation and neurotoxic effects using multiple markers together with HMGB1. Given the known role of HMGB1 in neurodevelopmental processes, it is possible that elevated HMGB1 levels could contribute to the pathogenesis of ADHD. However, due to the cross-sectional design of this study, we cannot establish a causal relationship. Therefore, while this remains a plausible hypothesis, further longitudinal studies are required to investigate the potential causal role of HMGB1 in the development of ADHD. Despite the exclusion of comorbid psychiatric disorders in our study, the effect of psychosocial stress levels, which were found to affect serum HMGB1 levels, could not be evaluated. Therefore, it is important to consider these limitations in future studies.

## Availability of Data and Materials

The datasets used and analyzed during the current study available from the corresponding author on reasonable request.
